# Impact of perioperative anticoagulation management on free flap survival in reconstructive surgery: a retrospective analysis

**DOI:** 10.1186/s12871-025-02975-6

**Published:** 2025-02-26

**Authors:** Saeed Torabi, Remco Overbeek, Fabian Dusse, Sandra E. Stoll, Carolin Schroeder, Max Zinser, Matthias Zirk

**Affiliations:** 1 Department of Anesthesiology and Intensive Care Medicine, Faculty of Medicine, University Hospital Cologne, University of Cologne, Cologne, Germany; 2 Department for Oral and Craniomaxillofacial and Plastic Surgery, Faculty of Medicine, University Hospital Cologne, University of Cologne, Cologne, Germany; 3 Department of Plastic, Reconstructive and Aesthetic Surgery, Faculty of Medicine, University Hospital Cologne, University of Cologne, Cologne, Germany

**Keywords:** Free flap surgery outcome, Perioperative Anticoagulation management, Intraoperative heparin Administration, Postoperative PTT-Guided Anticoagulation, Microvascular Surgery Complications

## Abstract

**Background:**

Despite advancements in surgical techniques and perioperative care for free flap reconstructive surgery, concerns persist regarding the risk of free flap failure, with thrombosis and bleeding being the most common complications that can lead to flap loss. While perioperative anticoagulation management is crucial for optimizing outcomes in free flap reconstructive surgery, standardized protocols remain lacking. This study aims to investigate the role of anticoagulation and perioperative practices in free flap reconstructive surgery and their impact on surgical outcomes.

**Methods:**

This retrospective, single-center study included all adult patients undergoing free flap surgery from 2009 to 2020. Patients were retrospectively divided based on intraoperative (UFH or no UFH) and postoperative anticoagulation management (UFH only, Aspirin and UFH, Aspirin only). The relationship between anticoagulation protocols, PTT values, and flap survival was assessed.

**Results:**

A total of 489 free flap surgeries were included. Most flaps were taken from the upper extremity (49.5%), primarily for tumor-related reconstructions (85.7%). Flap loss occurred in 14.5% of cases, with a median time to flap loss of 3 days post-surgery. Intraoperative UFH (20 IU/kg) was administered to 63.6% of patients and significantly predicted flap survival (OR = 0.45, 95% CI [0.24, 0.82]). PTT values on day 1 post-surgery were significantly related to flap survival (P = 0.03), with each unit increase reducing the relative probability of flap loss by 5.2%. There was no significant difference in flap survival between patients treated with heparin alone and those treated with both heparin and aspirin. The small sample size in the aspirin-only group limited the statistical relevance of this subgroup.

**Conclusion:**

Our findings highlight the importance of intraoperative UFH and PTT-guided postoperative management in improving free flap survival. Standardized anticoagulation protocols are essential for enhancing outcomes in free flap reconstructive surgery.

## Introduction

Free flap transplantation has become a standard and reliable reconstructive procedure for addressing soft tissue and bone defects in head and neck surgery for trauma or cancer, achieving success rates of approximately 95% [1, 2]. To support these outcomes standardized perioperative care measures have been incorporated into Enhanced Recovery after Surgery (ERAS) protocols [3–5] with additional focus on intra-operative and post-operative physiological support [6–8]. Since the initial establishment of free flap reconstructive surgery in 1972 [9], various perioperative predictors have been analysed as crucial for successful free flap transplantation, including perioperative anticoagulation therapy [10–12].

Despite advancements in both surgical technique and perioperative care, concerns persist regarding the risk of free flap failure [11, 13, 14], with thrombosis and bleeding being the most common complications leading to flap loss [15, 16]. Although various therapy protocols have been proposed [15, 17–19], a standardized approach of perioperative anticoagulation management is still lacking [12]. Previous studies show contradictory results [20]: while some do not attribute any benefit to postoperative anticoagulant administration [20, 21], others demonstrate positive outcomes [18, 19, 22].

Perioperative thromboprophylaxis is particularly critical in head and neck microvascular reconstruction, where patients face a heightened risk of venous thrombosis [23, 24]. However, the choice of anticoagulant remains debated [1, 10, 15, 21, 22, 24], underscoring the need for further research to optimize management strategies for hypercoagulable states in microsurgical patients [4]. While success rates in microvascular free tissue transfer are high [1, 10, 16], the lack of consensus on antithrombotic agents highlights the need for evidence-based guidelines in this field.

This study aims to investigate the role of anticoagulation and perioperative antithrombotic practices in free flap reconstructive surgery. Our objective is to contribute to the ongoing discourse on standardized and optimized perioperative anticoagulation management in free flap surgery, with the ultimate goal of improving long-term outcomes.

## Materials and methods

This study is a retrospective, single centre study at the University Hospital Cologne in Germany. Included in this study are all adult patients who underwent microvascular flap transplantation at the Center for Dental, Oral, and Maxillofacial Medicine of the University of Cologne between the years 2009–2020. Patients were excluded in case of intra- or postoperative demise before flap healing.

### Ethical approval

The study protocol was approved by the institutional ethics committee of the University of Cologne. (Approval Nr. 24–1305)

### Data Collection and Review

Patient data for this study, including demographics, comorbidities, length of ICU / hospital length of stay and discharge information were retrospectively collected from the electronic hospital information system (ORBIS), OPS (operating procedure scores) codes and paper medical records using a standardized case report form. Data on diagnosis, defect localization, flap type, site and duration of surgery were also collected. Data collection encompassed a thorough review of intraoperative anesthesia protocols and postoperative patient records. Missing data were addressed as follows: patients with incomplete or missing critical information pertinent to the primary or secondary outcomes (e.g., details on intraoperative anticoagulation or postoperative PTT values) were excluded from the analysis. This approach was implemented to maintain the accuracy and reliability of the statistical models. Specifically, 140 cases were excluded due to insufficient documentation, resulting in a final dataset of 349 patients with complete records for analysis (Table 3). To assess the effect of intraoperative anticoagulation, patients were retrospectively divided into two groups based on whether heparin was administered during surgery. Additionally, all patients were retrospectively divided into three groups based on their postoperative anticoagulation regimen: 1) Heparin, 2) Heparin + Aspirin, and 3) Aspirin only (100mg/ day). The decision on intraoperative and postoperative anticoagulation therapy was made by the attending surgeon at the time and didn’t follow a given protocol. Postoperative administration of unfractionated heparin was initiated with an infusion pump (25000 IU/50 ml) at an initial dose of 500 IU/h, followed by PTT-controlled dosing every four hours for the first three days. Partial thromboplastin time (PTT) values for these first three days, along with the thrombocyte count, were also assessed (Table 4).

### Statistics

The collected patient data were initially entered into an Excel spreadsheet and numerically coded. IBM SPSS Statistics for Mac Version 27.0 (Statistical analysis software by IBM, SPSS Inc., U.S.A.) was used for statistical analysis. Categorical variables were examined for their frequencies and percentages, and metric variables for their means (standard deviation) and medians (interquartile ranges) depending on their distribution. Nominal parameters were examined for significance using cross-tabulations with Chi-square, Fisher's exact test or the Monte Carlo Simulation. Logistic regression analysis was performed on the categorical variables found significant in the cross-tabulations. Metric variables were analysed using the student’s t-test or the Mann-Whitney-U-Test. Metric variables were also examined for their influence on flap survival using logistic regression. The prerequisites for conducting logistic regression were examined using the Box-Tidwell method. Additionally, the models were checked for multicollinearity to prevent correlation among individual variables. Statistical significance was considered at *p*-value < 0.05.

## Results

### General findings

**Fig. 1:**
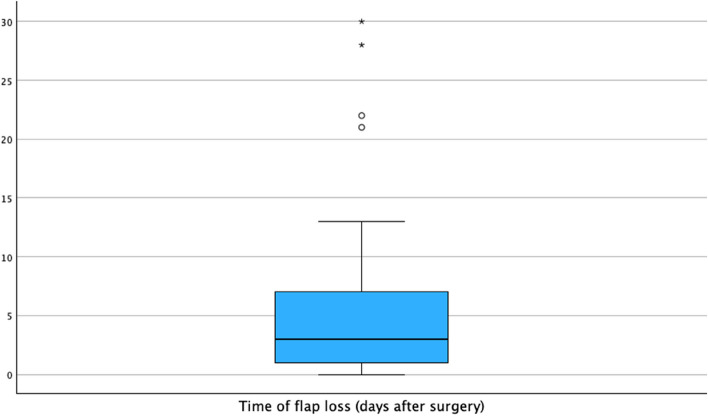
Time of flap loss (days after surgery)

From 2009 to 2020, a total of 489 free flap surgeries could be included in this study. Of all patients, 53,8% were male, and the median age was 62 years (IR 53.8–72 years). A total of 71 free flap surgeries resulted in flap loss (14.5%). Median time of flap loss was day 3 after surgery (Fig. 1). There was no significant relationship between age, sex, or comorbidities of the patient and flap loss (Table 1).

**Table 1:** Demographic data and comorbidities. PAD: peripheral arterial disease

Variable	Total	Flap loss	Flap survival	*p*
	*n* = 489	*n* = 71	*n* = 418	
Age (years)	62 (53.8–72)	61 (52–70)	62 (54–72)	*P* = 0.36
Sex (male)	263 (53,8%)	42 (59.2)	221 (52.9)	*P* = 0.33
**Comorbidities**	*(484)*	*(71)*	*(413)*	
Hypertension	195 (40.3%)	29 (40.9%)	166 (40.2%)	*P* = 0.92
PAD	28 (5.8%)	6 (8.5%)	22 (5.3%)	*P *= 0.32
Nicotine history	97 (20%)	15 (21.1%)	82 (19.9%)	*P* = 0.81
Diabetes mellitus	67 (13.8%)	8 (11.3%)	59 (14.3%)	*P* = 0.50

**Fig. 2:**
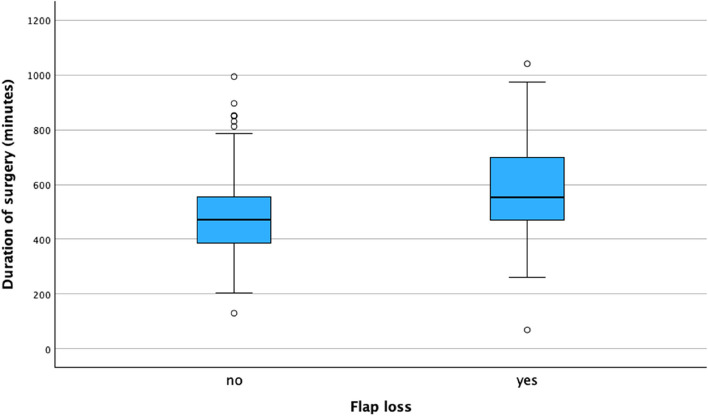
Duration of surgery (minutes) and flap loss

Most free flaps were taken from the upper extremity (49.5%), and the main reason for requiring a reconstructive flap surgery was a tumour (85.7%). The location of the defect was intraoral in 72.3% of patients (Table 2). The type of defect, localization of the defect, and origin of the flap were all significantly related to the probability of a perioperative flap loss (Table 2). Surgeries more often resulted in flap loss when the defect localization and the flap origin were in the lower extremity. Conversely, surgeries performed intraorally, and flaps taken from the upper extremity were more likely to result in flap survival. (Table 2). The relation between duration of the surgery and flap loss was also statistically significant (P < 0.001) (Fig. 2).

**Table 2:** Perioperative diagnosis, localization of defect, type of defect, origin of flap and time of surgery related to flap loss. The p-values marked with an * were calculated using the Monte-Carlo method

Variable	Total	Flap loss	Flap survival	*p*
	*n* = 489	*n *= 71	*n* = 418	
Diagnosis	*(489)*	*(71)*	*(418)*	
Tumour	419 (85.7%)	51 (71.8%)	368 (88%)	P < 0.01
Infection	15 (3.1%)	5 (7%)	19 (4.6%)	P = 0.06
Trauma	19 (3.9%)	4 (5.6%))	15 (3.6%)	P = 0.43
Other	35 (7.2%)	11(15.5%)	24 (5.7%)	P < 0.01
Localization of defect	*(488)*	*(71)*	*(417)*	
Intraoral	353 (72.3%)	43 (60.6%)	310 (74.3%)	
Head/Neck	50 (10.2%)	12 (17%)	38 (9.1%)	
Arm	7 (1.4%)	0 (0%)	7 (1.7%)	P* < 0.01
Leg	49 (10%)	16 (22.5%)	33 (7.9%)	
Thorax	29 (5.9%)	0 (0%)	29 (7%)	
Defect	*(488)*	*(70)*	*(418)*	
Soft tissue	377 (77.3%)	54 (77.1%)	323 (77.3%)	
Bone	2 (0,4%)	1(1.4%)	1 (0.2%)	P* = 0.42
Bone and soft tissue	109 (22.3%)	15 (21.4%)	94 (22.5%)	
Origin of Flap	(489)	(71)	(418)	
Abdomen	27 (5.5%)	1 (1.4%)	26 (6.2%)	
Arm	242 (49.5%)	25 (35.2%)	217 51.9%)	P* = 0.01
Leg	173 (35.4%)	40 (56.3%)	133 (31.8%)	
Thorax	47 (9.6%)	5 (7%)	42 (10%)	
Time of surgery (minutes)	492.5 (144.9)	579.4 (183.8)	477.1 (131)	P < 0.01

### Influence of anticoagulation on flap survival

63,6% of our patients received intravenous unfractionated heparin intraoperatively (20 IU/kg). There was a significant relationship between flap survival and the administration of heparin during surgery (P=0.01) (Table 3). The binomial logistic regression model was also statistically significant, χ^2^(*1*) = 7.70, p = 0.01, with Nagelkerke's R^2^ = 0.04. The classification accuracy was 85.9%. The use of intraoperative heparin significantly predicted flap survival (p = 0.01) with an odds ratio (OR) of 0.45 (95% CI [0.24, 0.82]).

The low Nagelkerke's R^2^ values observed in our logistic regression models indicate that while the identified variables (intraoperative heparin administration, PTT values on postoperative day 1) show statistically significant associations with flap survival, they explain only a small proportion of the variability in the outcome. This suggests that other unmeasured factors, such as surgical technique, patient-specific characteristics may also play a critical role in determining flap survival.

There was no significant difference in flap survival between patients treated with heparin alone and those treated with both heparin and aspirin. Aspirin without additional heparin was used in only three patients, with flap loss occurring in two of these cases.

**Table 3:** Intraoperative and postoperative anticoagulation therapy and influence on flap survival

Anticoagulation regime	Total	Flap loss	Flap survival	*p*
	*349*	*49*	*300*	
Intraoperative Heparin	222 (63.6%)	23 (46.9%)	199 (66.3%)	P = 0.01
Postoperative anticoagulation	*342*	*51*	*291*	
Aspirin	3 (0.3%)	2 (3.9%)	1 (0.3%)	P = 0.06
Heparin	261 (74.8%)	38 (74.5%)	223 (76.6%)	P = 0.74
Aspirin + Heparin	67 (19.6%)	10 (19.6%)	57 (19.6%)	P = 0.99

PTT values on the first day after surgery were significantly related to flap survival (P = 0.03). PTT Values on days two and three were lower in patients who experienced flap loss, though these differences were not statistically significant (Table 4). Logistic regression analysis for day one values indicated that the model (χ^2^(*1*) = 6.06, p = 0.01, Nagelkerke's R^2^ = 0.02, classification accuracy = 85.7%) was statistically significant. For each unit increase in the pTT value on day one, the relative probability of flap loss decreased by 5.2%. There was no significant correlation between thrombocytes count and flap survival (Table 4).

**Table 4:** Postoperative laboratory values. PTT: Partial thromboplastin time (second)

Variable	Total	Flap loss	Flap survival	*p*
	n = 489	n = 71	n = 418	
pTT day 1 (sec.)	31.2 (7.7)	29.3 (5.5)	31.5 (8.0)	P = 0.03
pTT day 2 (sec.)	35.5 (9.3)	34.6 (8.7)	35.4 (11.4)	P = 0.59
pTT day 3 (sec.)	31.3 (7)	30.6 (7.3)	33.0 (8.7)	P = 0.06
Thrombocytes day 1 (10^9^/L)	192.6 (69.7)	199.0 (83.5)	190.9 (67.3)	P = 0.38
Thrombocytes day 2 (10^9^/L)	161.8 (57.5)	169.3 (69.5)	163.5 (56.9)	P = 0.49
Thrombocytes day 3 (10^9^/L)	158.3 (57)	165.9 (58)	164.7 (61.4)	P = 0.88

## Discussion

### Summary of results

Our study demonstrated that the application of intraoperative heparin had a positive impact on free flap survival. Moreover, no additional benefit from the combination of heparin and aspirin could be detected. Prolonged PTT levels on the first postoperative day were associated with increased rates of flap survival whereas prolonged PTT levels on the second or third postoperative day were not associated with a better flap outcome. All these findings were independent of age, BMI and preexisting comorbidities.

However, we note that while intraoperative heparin uses and day-one PTT values are statistically significant in relation to flap survival, the low Nagelkerke's R^2^ values may indicate a weak relationship to flap survival in logistic regression analysis. Despite the low R^2^ values, the clinical relevance of our findings should not be underestimated. The statistically significant associations observed highlight key actionable factors that can guide perioperative management, such as the importance of intraoperative heparin administration and PTT-guided postoperative monitoring.

Our study also found that free flap reconstruction of the lower extremity, compared to the upper extremity or enoral sites as well as an extended duration of the surgery was associated with a higher chance of flap loss.

Contrary to our findings, Dawoud et al. [16] showed that anticoagulation therapy led to increased flap loss. However, this meta-analysis, which included eight retrospective studies that often involved heterogeneous patients with different anticoagulation therapy protocols, should be critically evaluated. Barton’s systematic review further illustrates the lack of consensus regarding anticoagulant protocols [10]. The most commonly used agents for antithrombotic therapy were aspirin [18, 25–27] and low-dose heparin [2, 18, 19, 28].

Preoperative factors such as age and gender were not associated with flap loss in our patient group, a finding corroborated by other studies [11]. Similarly, preexisting conditions such as arterial hypertension, peripheral artery disease (PAD), and diabetes mellitus showed no significant adverse impact on flap survival, consistent with previous studies [11, 29, 30]. More decisive for therapeutic success are the localization of the defect and the origin of the flap. Consistent with widely accepted views in the literature, intraoral defects with radial forearm flaps showed the best outcomes [24, 30, 31].

### Intraoperative management

In our patient cohort, the intraoperative administration of unfractionated heparin at a dose of 20 IU/kg showed a significant advantage in terms of flap survival. A European study by Rendenbach et al. demonstrated the widespread intraoperative administration of heparin following free flap harvesting, although the dosage varied significantly, ranging from 500 IU to 10,000 IU [12]. Other authors recommend the administration of aspirin intraoperatively and its continuation for three days postoperatively [18]. However, given a recent meta-analysis that found no benefit from aspirin, this practice should be critically evaluated [27].

Another crucial parameter affecting surgical outcome is the duration of surgery [29]. The prevailing opinion is that the duration of surgery should be as short as possible [11, 30, 32]. Chang et al. observed a negative effect on fibular flaps when the ischemia time exceeded 4 hours [32]. In 2016, Fichter et al. published a series of 8 patients with fibula grafts, demonstrating a success rate of over 96% following extracorporeal perfusion to reduce ischemia time [33].

Patients' body temperatures were continuously monitored using temperature probes, and active warming was achieved through the use of a Bair Hugger warming system. Additionally, pre-warmed intravenous fluids were consistently used during fluid therapy to minimize the risk of hypothermia, particularly given the prolonged duration of these surgeries. These measures are critical in preventing hypothermia-induced coagulopathy, which could negatively impact flap survival.

### Postoperative management

In literature, Rothweiler et al. divided complications into early and late phases, defining the first 72 h postoperatively as the critical phase. In our study, complications and flap loss peaked on the third postoperative day, also indicating a critical period within the first 72 hours [18]. Other authors have made similar suggestions regarding early postoperative management [34, 35]. Rothweiler recommends continuing aspirin (100 mg/day) at least during the early postoperative phase following an intraoperative loading dose of 300 mg and views UFH critically when administered at higher initial doses [18]. A recent meta-analysis demonstrated that the administration of aspirin has no direct effect on flap survival [27]. Our study also showed similar results, with no significant difference in flap survival between patients treated with heparin alone and those treated with both UFH and aspirin (100 mg/day). Therefore, the risk–benefit assessment regarding bleeding complications following the administration of aspirin should be carefully considered.

Contrary to this, we recommend intraoperative administration of UFH (20 IU/kg) instead and continuation of postoperative therapy with PTT-controlled UFH to minimize bleeding risk. While the postoperative administration of heparin has also been recommended by other authors [2, 19, 22, 28, 36], further analysis is needed to determine the optimal PTT target values for therapy management. While Rotweiler et al [18] aimed for a PTT of 60–80 seconds and noted increased bleeding rates with higher doses of UFH, we suggest targeting a PTT of 40–60seconds [2, 19, 22, 28, 36].

A comparable alternative to avoid frequent PTT monitoring is the administration of enoxaparin. Karimi et al [15]. achieved similarly favourable results with a postoperative enoxaparin dosis of 40 mg/day, showing a short-term success rate of 100. However, their study included only 30 patients. Sievert et al compared postoperative UFH and LMWH, finding comparable hematoma and revision rates with both heparin options [19]. Similarly, a meta-analysis by Dawoud et al. demonstrated comparable outcomes for postoperative administration of UFH and LMWH [16]. Another risk factor for thrombosis is perioperative thrombocytosis, particularly in trauma patients [37]. Kalmar et al [38] identified platelet counts over 250 k/mcL as a risk factor for flap failure in breast reconstruction [38]. We found no significant correlation between platelet counts during the first three postoperative days and flap failure.

In summary, although various studies have introduced different strategies for managing anticoagulation therapy, a lack of consensus and clear guidelines persists. Additionally, patient-specific factors, particularly in an aging population, must be considered, such as anticoagulation therapy for patients with pre-existing atrial fibrillation.

We also observe a continuously evolving approach to postoperative monitoring of transplanted flaps. Wax et al. demonstrated that intraoperative Doppler ultrasonography enhances the detection of immediate vascular complications, thereby reducing the need for subsequent revisions [39, 40]. Zinser et al. further introduced advanced ultrasound techniques to assess the perfusion of already implanted flaps, indicating a significant advancement in postoperative care [41, 42].

it is important to highlight that several factors contribute to the success of free flap surgeries and flap survival, as demonstrated in various studies [14, 43]. These factors include the duration of the surgery, preoperative radiation, the type of flap harvested, and the anastomosis technique employed. While our study focused primarily on anticoagulation, it is important to recognize that not only thrombotic events, which may lead to ischemia, and anastomotic insufficiencies are critical for flap viability, but haemorrhagic complications also play a significant role in the overall outcome.

In conclusion, our findings emphasize the importance of a standardized perioperative anticoagulation protocol in free flap surgery. Specifically, the intraoperative administration of unfractionated heparin (UFH) at a dose of 20 IU/kg was shown to significantly improve flap survival and should be incorporated into routine practice. Postoperative management using PTT-guided UFH therapy, targeting a range of 40–60 seconds during the first three days, offers an effective approach to balancing the risks of thrombosis and bleeding. Additionally, the absence of a significant benefit from combining UFH with aspirin suggests that aspirin may not be routinely necessary, simplifying the anticoagulation regimen for most patients while maintaining safety and efficacy.

### Limitations

In our study, we aimed to analyse perioperative data from our patient cohort regarding anticoagulation therapy, addressing the current data gap and lack of guidelines for postoperative anticoagulation in patients undergoing flap transplantation.

Limitations of our study include its retrospective nature and a highly diverse patient cohort with various flap types. Another limitation of our study was the absence of data for some patients, limiting the final analysis to 349 patients. Additionally, the sample size in group three, which received aspirin only, was small, preventing significant statistical findings. It is important to note that, due to lack of data such as antithrombin III levels, a complete picture of postoperative anticoagulation monitoring cannot be presented here. Furthermore, due to missing data, complications such as bleeding or thrombosis could not be adequately assessed.

Another major limitation is the decision for anticoagulation regime, which was made by the attending surgeon based on individual preference and patient characteristics and the quality of anastomosis without a clear protocol. Given the discrepancies in published studies, we recommend a prospective multicenter study for future research.

## Conclusions

Despite the lack of consensus regarding perioperative anticoagulation therapy, we conclude that anticoagulation is essential for free flap preservation and surgical success. Based on our experience, a combined anticoagulation approach, with intraoperative heparin at 20 IU/kg followed by postoperative unfractionated heparin for the first three days, using a PTT-guided therapy with a predefined target range of 40–60 seconds, as implemented in our clinic, represents an effective option for postoperative management. We were unable to identify any significant benefit from the supplementary administration of aspirin postoperatively.

## Data Availability

No datasets were generated or analysed during the current study.
